# Not so elementary – the reasoning behind a medical diagnosis

**DOI:** 10.15694/mep.2019.000234.1

**Published:** 2019-12-17

**Authors:** Joanna Sooknanan, Terence Seemungal

**Affiliations:** 1 Independent Researcher; 2 The University of The West Indies

**Keywords:** medical diagnosis, clinical reasoning, abductive logic, deductive logic, inductive reasoning

## Abstract

This article was migrated. The article was marked as recommended.

Making a diagnosis is a complex process involving incomplete yet dynamic information. At no other time in the history of medicine has information been so readily available and accessible resulting in a greater need for clarity of thinking. A clear understanding of the underlying reasoning processes involved is necessary so as to avoid misdiagnosis and to avoid unnecessary often costly and time-consuming tests. This article explores the main reasoning processes inherent in the making of a diagnosis - deduction, induction and abduction.

## Introduction

With all the medical tests and equipment available in our increasingly technological age, the inexperienced might be forgiven in thinking that making a clinical diagnosis is as simple as shaking the proverbial medical caduceus and ordering the appropriate medical test or procedure. An illusion further heightened by the popularity of medical dramas and their predisposition for diagnosing diseases with a dramatic denouement based on some sophisticated medical test. However, real life is not as obliging - at least half of the world’s population is unable to access essential health services (World Bank, 2017). On the other hand, clinicians with unrestricted access to tests and equipment may encounter emergency and acute care situations whereby they must act with incomplete information while awaiting test results. This means that the ability to make diagnoses without the benefit (crutch?) of sophisticated tests is a vital skill to develop and foster in the any doctor who must be prepared for all contingencies.

An investigation into the diagnostic process leads to some insight into how this can be achieved. In its simplest form, the diagnostic process entails matching information regarding symptoms and signs with an illness. This information is usually obtained from a history and physical examination of the person seeking medical care which might be supplemented by diagnostic procedures. The practitioner then synthesizes all this information based on his knowledge and experience so as to make decisions about prognosis and treatment options ( Groves, Scott and Alexander, 2002). Clinical judgment, medical logic, clinical method, clinical reasoning, clinical action, medical reasoning, problem solving, decision making and critical thinking and medical thinking are some terms used to describe this cognitive process.

Traditional teaching in medical school does not formally introduce reasoning and logic ( Trimble and Hamilton, 2016). Instead, clinical reasoning is introduced as part of the clinical experience in “a piecemeal fashion” ( Fuks, Boudreau, and Cassell, 2009) during ward rounds and is dependent on the nature of the clinical cases and the quality of supervision ( Croskerry *et al*., 2017). As any good clinician can attest, this reasoning process is not independent of everyday experience. The ability to think logically and critically is a skill developed in daily life from an early age. Thus biases and errors may have unconsciously transferred into the diagnostic process due to habitual, erroneous thinking. For this reason, understanding the reasoning processes by which inferences are made is important in helping clinicians improve their diagnostic skills ( Lawson and Daniel, 2011).

To begin the journey into the thinking processes involved, we use the analogy of the doctor as a detective (Peschel and Peschel, 2005). Just like a detective, a doctor is presented with clues and evidence and uses this information to identify an illness and thus solve the case. It is no coincidence that one of the most well-known of all fictional detectives, Sherlock Holmes, was the creation of a medical doctor Sir Arthur Conan Doyle. Holmes’ methodical investigative practices and complementary thought processes using his “little grey cells” (à la Hercule Poirot) share a remarkable similarity with the diagnostic process. Both groups have the same goal in mind and use similar methods to achieve this end - reasoning and logic.

## The reasoning processes

In a Star Trekesque Utopian world, making a diagnosis would be a simple matter. A doctor would scan a patient with a device (a tricorder) and a disembodied voice would reveal the diagnosis with certainty. It is unfortunate (or fortunate depending on your perspective!) that in our reality this device remains an elusive goal so that the clinician must rely on signs and laboratory results (objective facts) and symptoms (subjective facts) to establish a diagnosis. For the neophyte medical student, the process of arriving at a diagnosis may appear to be somewhat intimidating and mysterious. Yet, when viewed from a reasoning perspective, this is not one that is unfamiliar to students. It is very similar to that used in their daily lives when making a decision and choosing one option over others, in developing an argument to justify a claim or when seeking possible explanations. For example, to account for poor performance in an examination or other activity or to determine why a computer program or app is not working.

An argument is a collection of statements (premises), some of which are used in support of another (the conclusion) ( Fee and Belland, 2012). A typical example of an argument is - If you want to be smarter (more beautiful, lose weight etc.) you should take Product X. You want to be smarter (more beautiful, lose weight etc.). Therefore, you should take Product X. The first two sentences here are the premises of the argument, and the last sentence is the conclusion.

In medicine, reasoning processes are generally used in conjunction with an effect, with the goal of determining possible causes/ explanations/diagnoses. This article examines the three types of reasoning frequently employed by clinicians - deduction, induction and abduction ( Arocha, Wang and Patel, 2005; Cooper and Frain, 2016). We shall discuss each of these in turn and then examine how each is inherent in the diagnostic reasoning processes of an experienced clinician.

## Deductive Logic

**Figure 1.**
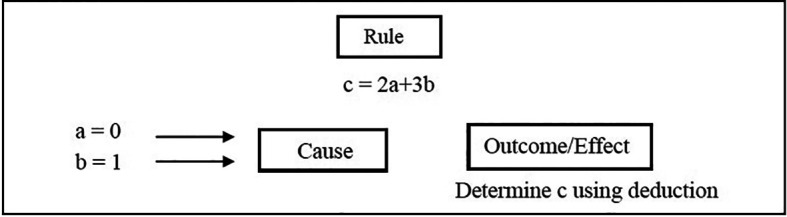
The process of deduction.

A familiar use of deductive logic in everyday thinking is shown in Illustration 1. Central to this reasoning process is a general rule relating a cause to an effect. This is then used to reason “down” to certain outcomes/effects. For the mathematically inclined, Figure 1 shows that deductive reasoning involves a rule which is used to reach a conclusion (here c = 2(0)+3(1) = 5). This form of reasoning is an example of ‘explicative reasoning’ where the conclusions made are nothing new but already incorporated in the premises.

Although many may openly scoff at the absurdity of the premise in Illustration 1, this example highlights a major weakness of deductive reasoning - though the conclusion is logically sound, if the rule is incorrect the process is flawed.

Illustration 1:

Example of Deductive Logic in everyday experience

*Premise/Rule*: Every time I wash the car, it rains

*Cause*: I am washing the car today

*Effect/Outcome*: It will rain today.

Deductive reasoning assumes that the basic law from which you are arguing is correct and thus applicable in *all* cases. The difficulty with depending solely on deductive reasoning skills is that diseases with pathognomonic features are not common so that the same symptom may occur in different diseases. Also, the same disease may have a profile of symptoms that may present in quite idiosyncratic ways in patients.

An example familiar to the medical student is shown in Illustration 2. Here asthma is diagnosed on the basis that wheezing is characteristic of asthma. However, the discerning medical student may wonder if this generalisation is true. While many persons who wheeze have asthma, wheezing is also present in acute bronchitis, tumors, chronic obstructive pulmonary disease and in other diseases such as bronchiolitis and even in cardiac failure. In the example above, though the reasoning is valid, since the rule is inadequate, application of this process will lead the practitioner to error in some cases of wheeze - with potentially detrimental consequences. Further, we are aware that though this example may seem trite, even experienced practitioners have been caught out like this simply because they did not recognize the error in the reasoning process employed or more precisely they had not realized that they were using the wrong reasoning process. In this case deductive reasoning based on the wrong premise leads to the wrong conclusion. In fact, because the cause of wheeze is many fold, deductive reasoning cannot be applied here.

Illustration 2:

A case of wheeze

Mr X, a 25 year old male patient, presents to his doctor with wheezing. What is the diagnosis?

*Rule*: All persons who wheeze have asthma.

*Cause*: Mr X is wheezing

*Outcome/Effect*:Mr X has asthma

## Inductive Reasoning

**Figure 2.**
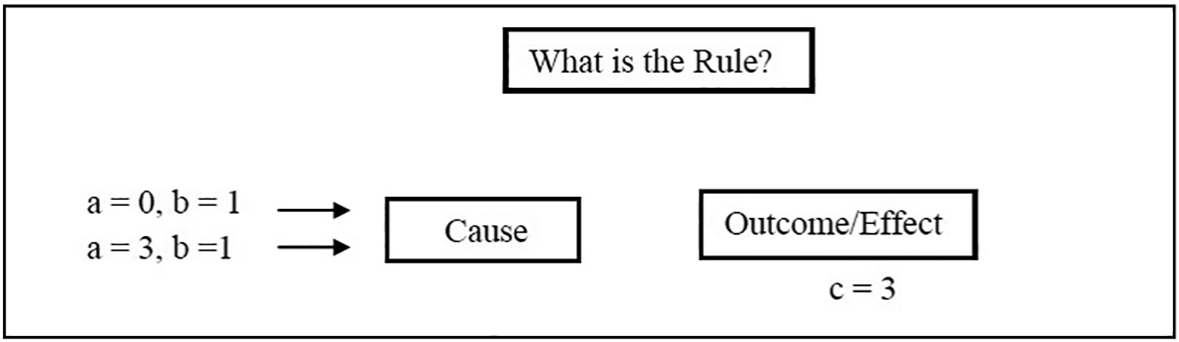
The process of induction.

In contrast, inductive reasoning uses specific examples to draw general conclusions. It attempts to find patterns in observations and measurements and then proceeds to infer a generalized rule that is likely, but not certain as shown in Figure 2. This general rule must be determined based on the causes and effects. An example with which many may be familiar plays on superstitious beliefs. If a student performs well in an exam when using a certain pen, he may consider that the pen brought him luck. If future successes (outcome) happen whenever that pen (cause) is used, then this belief may be reinforced and the pen may be considered as “lucky” (rule).

Inductive reasoning may be considered as reaching a conclusion based on the similarity with past experiences or observations. It is a dynamic process whereby conclusions are drawn from experiences resulting in the formation of beliefs, which are updated and refined when necessary based on emerging evidence - Bayesian updating. There are several types of thinking that are collectively referred to as inductive reasoning: generalization, analogy, causation, authority and parallel case thinking ( Bartha, 2010) and pattern recognition.

Many clinicians utilize inductive reasoning in the form of clinical pattern recognition based on experience and personal knowledge as part of their repertoire of cognitive diagnostic tools (Ridderikhoff, 2012). This entails the recognition of new problems as similar or identical to ones already encountered and so allows for quick decision making (Coderre *et al*., 2013; Simmons, 2010).

For example, suppose a clinician is faced with a patient with sudden onset left-sided chest pain. Upon completing the history and physical examination a chest radiograph is requested and this shows an area of extra air in the left lung - a pneumothorax. A reasonable inference is that the pneumothorax is the cause of the chest pain. However, there are over 100 causes of chest pain (e.g. myocardial infarction, aortic dissection, pleurisy, pneumonia, fractured rib, myositis, cystic lung disease, pneumothorax amongst many other causes), so why choose this one as worthy of consideration? The answer is that the clinician related the events by probability through inductively considering all of the cases within his or her experience and decided that this is by far the most likely cause. So the clinician infers that the pneumothorax is the (likely) cause of the chest pain in this patient. Let us consider the opposite - suppose this clinician had never learnt about or seen a case of pneumothorax. This would not invalidate the diagnosis however using inductive reasoning, the clinician would not have come to diagnosis. The clinician may then have diagnosed cystic lung disease or pleurisy - both of which would be incorrect.

Induction yields discoveries that are probable, but not proven. This example highlights the subjective nature of this method of reasoning which may lead to conflicting opinions by different doctors. Uncritical use may result in the clinician disregarding “other rare causes of the combination of features” presented ( Willis, Beebee and Lasserson, 2013). The clinician must concurrently consider “multiple competing hypotheses by weighing their relative probabilities” ( Croskerry *et al.*, 2017).

Unlike deductive reasoning, inductive reasoning can increase human knowledge leading to new theories. However, another word of caution is necessary when using this reasoning process. This method relies on a “large enough” number of observations to generalize - the user should always bear in mind that the outcome is likely but not certain (as distinct from the deductive reasoning process). For example, studies have demonstrated the analgesic properties of paracetamol so that by induction we can infer that taking paracetamol is likely to alleviate a headache. The difficulty inherent in this reasoning process is highlighted by its description as “the skeptical problem about the future” (Hacking, 1975) - the future does not always resemble the past and there may be outliers. This means that paracetamol may not relieve a severe headache.

## Abductive Reasoning

Though the modus operandi of the legendary fictional detective Sherlock Holmes with his uncanny ability to surmise plausible details about a person or scene has been associated with deduction, he actually uses a method of reasoning called abduction ( Carson, 2009; Steiff, 2011). Abductive reasoning typically begins with an incomplete set of observations and proceeds to the likeliest possible explanation - it infers the precondition “a” from the consequence “b”. It differs from the other reasoning processes - whereas deductive reasoning deals with certainty and inductive reasoning with probability based on data, abductive reasoning entails a best guess approach based on a limited set of information. Since this is best guess approach the clinician is aware of the limitations of diagnostic technique used.

Since there is always the possibility of error, it allows for alternative explanations/hypotheses. For example, suppose you call a friend on the phone and there is no answer. In searching for an explanation, you may adduce that she is avoiding you. Alternatively, you may consider other possible explanations - maybe she did not hear the phone or you dialed the wrong number. This is the essence of abductive thinking.

**Figure 3.**
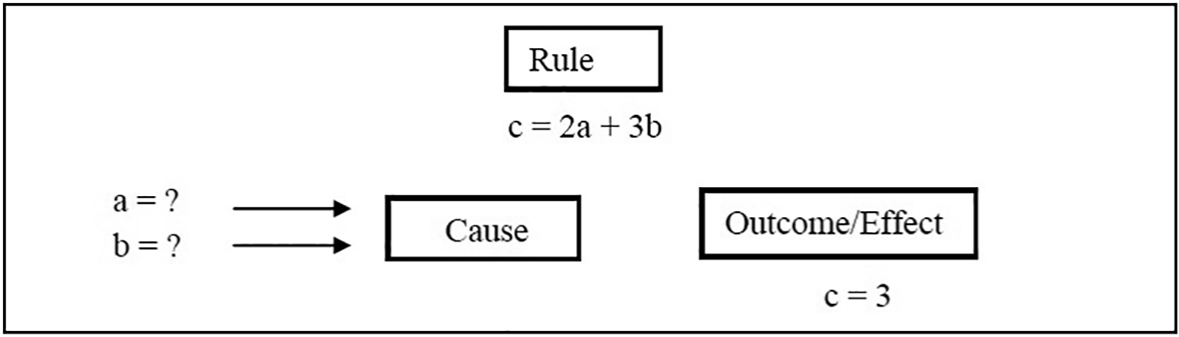
The process of abduction.

For the mathematically inclined, Figure 3 shows that while abductive reasoning involves a rule and an effect, the cause must be determined. Analogous to the multiple competing hypotheses associated with abduction, it is noted that there may be multiple pairs of values for a and b such as (a,b) = {(0,1), (3,-1),(-9 ,7)}.

Deductive and inductive reasoning processes by themselves are inadequate to describe what takes place during the initial stage of the diagnostic process where the clinician must come up with possible diagnoses as they cannot contribute to the development of explanatory theories ( Mirza *et al.*, 2014; Eriksson and Lindström, 1997; Haig, 2008).

Abduction is used to generate possible explanations/hypotheses for incomplete observations, surprising facts or puzzles early in the diagnostic process. It acts as a precursor to the next stage in the diagnostic journey - deduction. Deductive reasoning is used to determine what tests need to be conducted to explore the consequences of hypotheses ( Eriksson and Lindström, 1997). Here, a hypothesis is assumed to be true and the clinician must decide on the tests necessary to confirm or reject it. If the predicted and observed results match, thenthe hypothesis is supported but not confirmed since other tests related to alternative hypotheses may show similar results. Otherwise, it is rejected ( Lawson and Daniel, 2011). This is the inductive phase which compares the claims of the hypothesis with the observable facts. Each confirmatory or negative test result leads to in an increased confidence in the final diagnosis.

Subsequent confirmation of our hypothesis - by observations, experiments or other future evidence - makes it even more well-confirmed. Though abduction and deduction contribute to our conceptual understanding of a phenomenon ( Hausman, 1997) the logic of induction provides empirical support by justifying the hypothesis with empirical data (Staat, 1993).

When selecting hypotheses, medical practitioners generally use the aphorism “when you hear hoof beats, think horses, not zebra” or “common things are common” as a guide. In medicine, a zebra is a disease that is often studied but seldom seen - such as the Crigler-Najjar syndrome or Ehlers-Danlos syndrome or Dubin-Johnson syndrome. Despite their low probability of occurrence, their exotic nature makes them memorable to the novice who may be apt to see these “zebras” everywhere instead of first focusing on common conditions and diseases (horses). For example, a patient may be suffering from jaundice, and though a viral infection is the most likely cause, Dubin-Johnson syndrome may be initially considered by the beginner. This does not mean that “zebra”-type diagnoses have no value. Instead they must be held in mind until common causes are ruled out re Occam’s Razor where ‘among competing hypotheses, the one with the fewest assumptions should be used.’

It would be remiss of us not to include a subtle example of abductive reasoning which the novice may not consider. To do so, we turn again to the master crime solver Sherlock Holmes for inspiration in a case involving the disappearance of a famous racehorse. In that case, the absence of barking from the watchdog revealed the criminal to be the horse’s trainer with whom the dog was familiar. This bit of reasoning a “negative fact” or an absent expected fact is one that has been developed by experienced doctors who “have the ability to spot possible inconsistencies among the clinical, instrumental, and laboratory examinations, considering not only what is present but also what is missing” ( Rapezzi, Ferrari and Branzi, 2005).

Individually, knowledge of each of these reasoning processes is a useful tool in the diagnostic toolkit, but how are they holistically applied to an actual diagnosis?

## An unusual SOB story – The case of the clandestine sarcoidosis

Consider the puzzling case of a 42-year-old male who presented with epigastric pain and a one-day history of sudden onset shortness of breath ( Sandy *et al.*, 2011).

Using abductive reasoning, the doctors hypothesized possible causes of pain and SOB as pancreatitis, pneumonia, pulmonary embolism, myocardial infarction (heart attack). They then tested these theories individually using deductive logic. For example, if the patient had pancreatitis then a blood test would show elevated amylase or lipase level. The result of the test was negative so by induction the doctors ruled out pancreatitis and a similar negative blood test and ECG allowed them to rule out a myocardial infarction. A chest scan showed a pulmonary embolism - using inductive reasoning the doctors concluded that on a balance of probabilities, it was likely that the cause of the shortness of breath was a pulmonary embolism. However, the only identifiable risk factor for this pulmonary embolus was obesity. Further, there was also ground glass shadowing on the CT chest scan - an unusual accompaniment of pulmonary embolus, as the patient improved this could easily have been ignored. However, armed with a dose of healthy curiosity, his doctors decided to investigate further. Coincident investigations then revealed the presence of sarcoidosis. The doctors concluded that this was the cause of the pulmonary embolism and further postulated by induction that sarcoidosis is a cause of pulmonary embolism. A literature search then revealed that there were only six such published cases in the world thus contributing to the body of medical knowledge of rare complications of sarcoidosis.

## Discussion

By themselves knowledge of these reasoning processes is useful, but how are these skills developed? Certainly most people - yet alone doctors ( Norman *et al.*, 2009) - are not consciously aware when they use these modes of reasoning, though they are innate in everyday decision making. What appears to be lacking in their “intellectual” doctor’s bag of tricks, is the judicious application of logical and rational thinking skills to the diagnostic process - a process that has been essentially invisible and seldom elucidated in medical school training.

The first formal exposure most doctors have to reasoning is via the Problem-based learning (PBL) approach used by many medical schools to help students develop their clinical reasoning skills ( Ju and Choi, 2017). This method combines elements of inductive and deductive reasoning into one scheme known as the hypothetico-deductive approach (Cooper and Frain, 2016). According to this model, early in the encounter with the patient, possible explanations (hypotheses) are generated based on clinical data and knowledge. These hypotheses are then used to guide further data collection aiming at either confirmation or refutation until a final hypothesis was accepted.

Perhaps because of their familiarity with this approach, this is commonly used by novices although mature clinicians are not immune to using it when facing an unfamiliar case outside of their area of expertise or a complex case ( Lawson and Daniel, 2011; Simmons, 2010).

While the hypothetico-deductive approach is useful in excluding some of the possibilities, a definite diagnosis cannot be made since it is not possible to ascertain whether all the possibilities/hypotheses were considered. It fails “to describe the initial phase of inquiry, which is related to the discovery of hypotheses” ( Mirza *et al.*, 2014).

In the problem-based learning curricula of medical schools, there can be an over emphasis on a hypothetico-deductive approach to problem-solving. This to the exclusion of and detriment to abductive reasoning. Abductive reasoning allows the linkage of phenomena with unusual causes and generates plausible explanations of issues at hand. The difference between these two approaches to problem-solving can lead to an over estimation of the importance of problem-based learning in the development of clinical reasoning.

At no other time in the history of medicine has information been so readily available. Yet, it has been estimated that 12 million Americans experience a diagnostic error each year, of which 40,000-80,000 are fatal ( Croskerry *et al.*, 2017). This is despite the rapid evolution of technology resulting in an abundance of medical tests as well as a rapid and easy access to information - essentially at a clinician’s fingertip.

Over-reliance on technology is both a strength and weakness in diagnostic medicine ( Rapezzi, Ferrari and Branzi, 2005). With the temptation of a surfeit of tests and available information, it is imperative that clinicians have an explicit understanding of the reasoning processes fundamental to a diagnosis. This is important so as not to subject the patient to unnecessary tests as well as to ensure a timely diagnosis.

## Conclusion

Making a diagnosis is a complex phenomenon involving incomplete, often extraneous dynamic information. Just as solving a mystery allows for the exercising of the “little grey cells”, a clear understanding of the underlying reasoning processes is necessary so as to avoid misdiagnosis and to avoid unnecessary often costly and time-consuming tests.

## Take Home Messages

• Making a diagnosis is a complex process involving incomplete yet dynamic information.
• The three types of reasoning frequently employed by clinicians are deduction, induction and abduction.
• Abduction is used to generate possible explanations/hypotheses early in the diagnostic process.
• Deductive reasoning is used to determine what tests need to be conducted to explore the consequences of hypotheses.
• The inductive phase compares the claims of the hypothesis with the observable facts.

## Notes On Contributors

JOANNA SOOKNANAN is an Independent Researcher with a PhD in Mathematics.

TERENCE SEEMUNGAL is the Dean of the Faculty of Medical Sciences, The University of The West Indies, St Augustine Campus with a MSc. in Mathematics. He has a special interest in the intersection of mathematics and medicine. He is programme director of postgraduate training in Internal Medicine.
