# Supplementary material for: Not so elementary – the reasoning behind a medical diagnosis
Source: MedEdPublish (2016). 2019 Dec 17;8:234. [Version 1] doi: 10.15694/mep.2019.000234.1 (PMC10712618; doi:10.15694/mep.2019.000234.1)
Supplement: Peer review history

# Reviewer report: Reviewer response for version 1

**DOI:** 10.21956/mep.19894.r30106

**Published:** 2020-01-03

**Authors:** Keith Wilson

**Affiliations:** 1 Dalhousie University

This review has been migrated. The reviewer awarded 4 stars out of 5 The authors of this paper really help the reader understand the complexities of making a diagnosis. They lead us through three main types of reasoning: deductive, inductive and abductive. How these types relate to the generation of diagnoses is clearly delineated. I found the writing style made this paper such a pleasure to read. The reader is led through successive examples that highlight the pros and cons of each type of reasoning in the context of making a diagnosis. The authors also conclude by applying the three reasoning types using an easy-to-understand example - it made me want more examples.In an era of increasing data, grounding our thinking in these types of reasoning models may indeed help steer away from medical error. This article is applicable to any educators involved in teaching diagnostic reasoning.

Reviewer Expertise:

NA

No decision status is available

# Reviewer report: Reviewer response for version 1

**DOI:** 10.21956/mep.19894.r30107

**Published:** 2019-12-29

**Authors:** P Ravi Shankar

**Affiliations:** 1 American International Medical University

This review has been migrated. The reviewer awarded 4 stars out of 5 This is an interesting manuscript. The authors have tried to examine the reasoning behind a medical diagnosis. I enjoyed the writing style of the authors and the frequent references to creative literature. Abductive reasoning was a new concept for me. The case study of clandestine sarcoidosis illustrates the concepts very well. An enjoyable and instructive read.

Reviewer Expertise:

NA

No decision status is available
